# MultimodalCNN-PD: a Parkinson’s disease diagnostics framework using multimodal convolutional neural network

**DOI:** 10.3389/fnagi.2026.1733075

**Published:** 2026-02-25

**Authors:** Tongle Zhi, Haonan Liu, Xuan Wang, Umar Muhammad Ibrahim, Chengjie Meng

**Affiliations:** 1 Department of Neurosurgery, Yancheng First Hospital Affiliated to Medical School of Nanjing University, Yancheng, China; 2 School of Artificial Intelligence and Automation, Huazhong University of Science and Technology, Wuhan, China

**Keywords:** Parkinson’s disease, early diagnosis, MRI, clinical metadata, multimodal CNN, deep learning

## Abstract

**Background:**

Parkinson’s disease (PD) is a prevalent neurodegenerative disorder that severely affects motor and cognitive functions. Early diagnosis, particularly during the prodromal phase, is critical for effective intervention.

**Methods:**

This study presents MultimodalCNN-PD++, a deep learning model that integrates Magnetic Resonance Imaging (MRI) with clinical metadata (including motor/cognitive assessments, demographic data, and genetic biomarkers) to enhance PD classification. The model employs a lightweight EfficientNetB0 backbone, Mobile Convolutional Block Attention Modules (Mobile CBAM), and an enhanced Meta-Guided Cross-Attention (MGCA++) mechanism. A three-stage hierarchical feature selection method identifies the most discriminative clinical features, while metadata is processed with BioClinicalBERT using Low-Rank Adaptation (LoRA).

**Results:**

Validated on the Parkinson’s Progression Markers Initiative (PPMI) dataset, the model achieved 97.5% accuracy in distinguishing Normal Control, prodromal PD, and diagnosed PD cases, with reduced parameters and computational costs. External validation on the OASIS-3 dataset confirmed robust generalizability (96.2% accuracy) despite demographic and acquisition protocol variations. Ablation studies highlighted the contributions of Mobile CBAM, MGCA++, hierarchical feature selection, and BioClinicalBERT-LoRA.

**Discussion:**

This framework sets a new benchmark for multiclass PD diagnosis, demonstrating strong potential as a clinically deployable AI tool for early detection and personalized management of neurodegenerative diseases.

## 1 Introduction

Medical progress has substantially extended global longevity, resulting in an accelerating demographic shift toward aging populations (Pereira et al., 2016). Demographic projections indicate that by 2100, approximately 90% of nations will transition into aged societies, with more than half achieving super-aged classification (Yang et al., 2025). This profound population transformation introduces significant burdens for healthcare infrastructure, particularly regarding the management of age-related neurodegenerative pathologies (Heuveline, 2022). Within this spectrum, Parkinson’s disease (PD) emerges as the second most prevalent neurodegenerative disorder, impacting over 10 million individuals globally (Marek et al., 2018). The fundamental pathophysiology of PD encompasses progressive deterioration of dopaminergic neurons within the substantia nigra pars compacta, manifesting in its hallmark motor symptoms including tremor, muscular rigidity, bradykinesia, and postural instability, alongside non-motor features such as cognitive decline and mood alterations (Bhagwat et al., 2018; Xue et al., 2018).

Disease progression traverses prodromal, early, and advanced stages, characterized by symptom intensification. Contemporary diagnostic approaches depend on neurological examinations, clinical interviews, and standardized motor assessment scales (including UPDRS, Hoehn and Yahr staging), supplemented by neuroimaging modalities such as MRI and DaT-SPECT for differential diagnosis. However, these traditional methods prove inadequate, demonstrated by early diagnostic error rates reaching 25%, underscoring an urgent requirement for advanced computational methodologies to improve diagnostic accuracy and support clinical decision-making (Qin et al., 2021). Traditional machine learning (ML) approaches, including support vector machines (SVM), random forest (RF) algorithms, and logistic regression (LR) models, have been extensively investigated for Parkinson’s disease (PD) diagnosis and progression forecasting (Li et al., 2024).

A fundamental constraint of these methodologies lies in their dependence on manually engineered features, representing a labor-intensive process demanding considerable domain specialization (Zhang et al., 2022). The emergence of deep learning (DL), particularly convolutional neural networks (CNNs), has resolved this limitation by facilitating automated, hierarchical feature extraction directly from medical imaging modalities, including MRI and DaT-SPECT acquisitions, thereby yielding enhanced diagnostic outcomes (Dahbour et al., 2021; Wang et al., 2023). However, conventional CNN architectures such as ResNet and VGG networks, while demonstrating excellence in image interpretation, suffer from excessive computational demands and parameter redundancy, limiting their deployment in resource-constrained clinical environments (Hwang and Kang, 2023). Recent advances in efficient neural network design, exemplified by EfficientNet’s compound scaling methodology, have demonstrated that careful balancing of network depth, width, and resolution can achieve superior performance with significantly fewer parameters (Xin and Li, 2023). Despite these architectural innovations, CNNs exhibit inherent limitations in modeling the temporal progression patterns and multimodal relationships critically for comprehensive PD assessment (Chen et al., 2020).

Within clinical practice, PD diagnosis extends beyond imaging-based assessment alone. Clinicians additionally evaluate patient medical histories, neuropsychological evaluation results, motor function assessments, and genetic information, generating heterogeneous and multimodal data collections (Tang et al., 2024). While imaging modalities including MRI and DaT-SPECT deliver essential diagnostic insights, supplementary metadata encompassing UPDRS scores, cognitive assessments (MoCA, MMSE), gait parameters, and genetic markers prove equally valuable for accurate diagnosis (Al-Azzawi and Al-Ani, 2024; Cao et al., 2023). Given the challenges in obtaining large-scale imaging datasets, effective utilization of multimodal information becomes critical for enhancing diagnostic precision. Although CNN-based architecture demonstrates superior performance in imaging data interpretation, most current multimodal frameworks encounter difficulties in effectively integrating heterogeneous data sources. Traditional attention mechanisms process modalities independently rather than leveraging dynamic, adaptive cross-modal interactions (Benítez-Andrades et al., 2022). Furthermore, the selection of clinically relevant features from high-dimensional metadata remains challenging, often relying on univariate statistical methods that ignore feature redundancy and complex nonlinear relationships (Acharya, 2024). These limitations constrain the clinical utility and cross-population generalizability of existing multimodal models for PD diagnosis.

To address the previously described challenges, this research introduces MultimodalCNN-PD++, an enhanced deep learning architecture that accomplishes precise three-class classification (Normal Control, Prodromal PD, Diagnosed PD) through integrated analysis of structural MRI and heterogeneous clinical data, incorporating textual reports, demographic information, genetic markers, and assessment scores with significantly improved computational efficiency (Teng et al., 2025; Zeng et al., 2024). The framework’s efficacy derives from an advanced feature aggregation mechanism engineered to capture sophisticated cross-modal relationships while maintaining interpretability through enhanced visualization techniques.

## 2 Related works

The synthesis of neuroimaging data with clinical textual information offers a comprehensive perspective on Parkinson’s disease (PD) heterogeneity, strengthening both diagnostic precision and prognostic modeling capabilities. Contemporary investigations have demonstrated that multimodal integration enhances performance beyond single-modality approaches (Benredjem et al., 2025). Numerous studies have explored deep learning methodologies for PD detection utilizing diverse data sources, including imaging, clinical assessments, and genetic information. However, the computational efficiency and parameter optimization of these models remain critical challenges for clinical deployment .

Earlier research predominantly concentrated on unimodal approaches, employing either neuroimaging or clinical features independently. Conventional machine learning algorithms, including SVM and random forests, were extensively applied to clinical datasets for PD classification. However, these methods encountered limitations in feature extraction and generalization performance. The introduction of deep learning architectures, particularly CNNs, revolutionized medical image analysis through automated feature learning capabilities. Multiple investigations have implemented CNN architectures, including ResNet, VGG, and DenseNet for PD diagnosis using MRI and DaT-SPECT imaging modalities, demonstrating enhanced classification performance compared to traditional approaches. Nevertheless, these architectures typically require millions of parameters and substantial computational resources, limiting their deployment in resource-constrained clinical settings. The development of efficient network architectures, particularly EfficientNet’s compound scaling approach, has demonstrated that careful optimization of network dimensions can achieve superior accuracy-efficiency trade-offs (Marek et al., 2018; Xue et al., 2018).

Recent developments have emphasized multimodal integration strategies to capitalize on complementary information from heterogeneous data sources. Attention mechanisms have emerged as fundamental components for effective multimodal fusion, enabling models to concentrate on relevant features across different modalities. The Convolutional Block Attention Module (CBAM) has been successfully implemented across various medical imaging applications, enhancing feature representation through spatial and channel-wise attention refinement. However, standard CBAM implementations introduce significant computational overhead through dense convolution operations. Mobile CBAM addresses this limitation by replacing standard convolutions with depth-wise separable convolutions, achieving 76% parameter reduction and 62% FLOPs reduction while maintaining attention effectiveness (Hwang and Kang, 2023; Xin and Li, 2023). Cross-attention mechanisms have proven effective for aligning information between distinct modalities, facilitating improved feature integration in multimodal learning frameworks. Traditional cross-attention implementations use fixed numbers of attention heads and simple concatenation-based fusion, lacking the adaptive capacity to balance modality contributions based on input characteristics.

Feature selection constitutes another critical component in multimodal PD diagnosis, particularly when integrating high-dimensional clinical metadata. Ensemble-based feature selection methods, combining multiple machine learning algorithms, have demonstrated robustness in identifying relevant clinical biomarkers. However, these approaches often overlook feature redundancy and inter-feature correlations, leading to suboptimal feature subsets. Recent work has emphasized the importance of redundancy-aware feature selection using mutual information theory and values for clinical interpretability. These approaches have been successfully applied in various medical diagnosis tasks, including neurodegenerative disease detection (Bhagwat et al., 2018; Qin et al., 2021).

For clinical text encoding, transformer-based language models have demonstrated remarkable success in capturing semantic relationships within medical documentation. Bidirectional Encoder Representations from Transformers (BERT) and its domain-specific variants, particularly BioClinicalBERT pre-trained on clinical notes, have shown superior performance in medical natural language processing tasks. However, fine-tuning these large models (110M parameters) for specific medical tasks requires substantial computational resources and annotated data. Low-Rank Adaptation (LoRA) has emerged as an efficient fine-tuning strategy, introducing trainable low-rank matrices while keeping pre-trained weights frozen, achieving 96% reduction in trainable parameters with minimal performance degradation (Cao et al., 2023; Tang et al., 2024).

## 3 Materials and methods

### 3.1 Multimodal learning framework for PD diagnosis

Let ${\left(X_{i},Y_{i}\right)}_{i=1}^{N}$. denote a set of training samples drawn from an underlying distribution *D*, where *X*represents the image domain, and *Y*corresponds to the associated ground-truth class labels. The goal of supervised deep learning is to learn a parametric function $F_{\theta }\,\left(X\right)\to \hat{Y}$that produces predictions $\hat{Y}$closely matching the true labels *Y*. Traditional approaches have predominantly relied on visual information to drive classification accuracy. However, auxiliary clinical variables, including demographic attributes such as age and weight, as well as cognitive and behavioral assessment scores from the Mini-Mental State Examination (MMSE), Montreal Cognitive Assessment (MoCA), Functional Activities Questionnaire (FAQ), and Neuropsychiatric Inventory Questionnaire (NPIQ) provide valuable diagnostic and prognostic insights (Shen et al., 2026; Teng et al., 2025). As a result, the principled fusion of clinical metadata with image-derived features becomes essential for improving predictive performance. To this end, the learning objective is reformulated as a multimodal mapping $F_{\theta }\,\left(X,X_{t}\right)\to \hat{Y}$, where *X*denotes imaging inputs and *X_t_*represents clinical metadata, aiming to effectively align and exploit complementary information from both modalities for more accurate and robust classification outcomes while optimizing computational efficiency (Sar et al., 2025).

### 3.2 Enhanced method overview

MultimodalCNN-PD++ is an enhanced multimodal deep learning architecture designed for accurate and computationally efficient Parkinson’s disease (PD) stage classification. This model jointly leverages neuroimaging data and clinical metadata through an improved Meta-Guided Cross-Attention (MGCA++) mechanism with dynamic adaptation capabilities. The framework consists of five key components, integrated into an efficient processing pipeline.

An EfficientNet-B0 backbone (5.3M parameters, 54.7% reduction from ResNet-18), enhanced with Mobile Convolutional Block Attention Modules (Mobile CBAM), is employed to extract and refine image representations through parameter-efficient depthwise separable convolutions, while selectively emphasizing salient spatial regions and informative feature channels. This backbone was selected due to its superior balance between expressive capability, computational efficiency, and robustness to overfitting, which is particularly critical when training data are limited (Safai et al., 2022). The compound scaling methodology of EfficientNet uniformly scales network depth, width, and resolution using a principled coefficient, achieving optimal accuracy-efficiency trade-offs (Benredjem et al., 2025).

A domain-adapted BioClinicalBERT model with Low-Rank Adaptation (LoRA) serves as the text encoder, efficiently converting clinical metadata including motor and cognitive assessment scores (UPDRS, MoCA), demographic information, Hoehn and Yahr staging, and genetic indicators (SNCA, LRRK2) into dense latent embeddings. The LoRA adaptation introduces trainable low-rank decomposition matrices (4.7M trainable parameters, 96% reduction) while keeping the pre-trained weights frozen, enabling efficient domain adaptation without full model fine-tuning.

A three-stage hierarchical feature selection pipeline identifies the most discriminative clinical variables through ensemble importance ranking, mutual information-based redundancy elimination, and SHAP-driven clinical validation. This systematic approach reduces the original 15 clinical features to an optimal subset of 5 features (UPDRS, Age, MoCA, Hoehn and Yahr stage, Weight) while minimizing inter-feature correlation and maximizing clinical interpretability.

The MGCA++ module performs adaptive multimodal feature fusion using dynamic multi-head attention with learnable head selection and gated fusion mechanisms. Unlike conventional cross-attention with a fixed architecture, MGCA++ dynamically determines the optimal number of attention heads (ranging from 2 to 6) based on input characteristics and employs a gating mechanism to balance the contributions of imaging and textual modalities. This enables more flexible and effective cross-modal alignment.

A fully connected classification head processes the fused multimodal representation, trained with a multi-component loss function combining focal loss (for class imbalance handling with γ = 2.0), triplet loss (for embedding discrimination with margin = 0.3), and consistency loss (for multimodal alignment). The training process employs advanced regularization techniques, including Mix-up data augmentation (α = 0.2 for images, 0.1 for metadata), label smoothing (∈ = 0.1), and stochastic depth (drop probability = 0.2) to enhance generalization. The final output assigns each subject to one of three classes: Normal Control (NC), prodromal PD, or diagnosed PD, with enhanced interpretability through Grad-CAM++ visualization.

**FIGURE 1:**
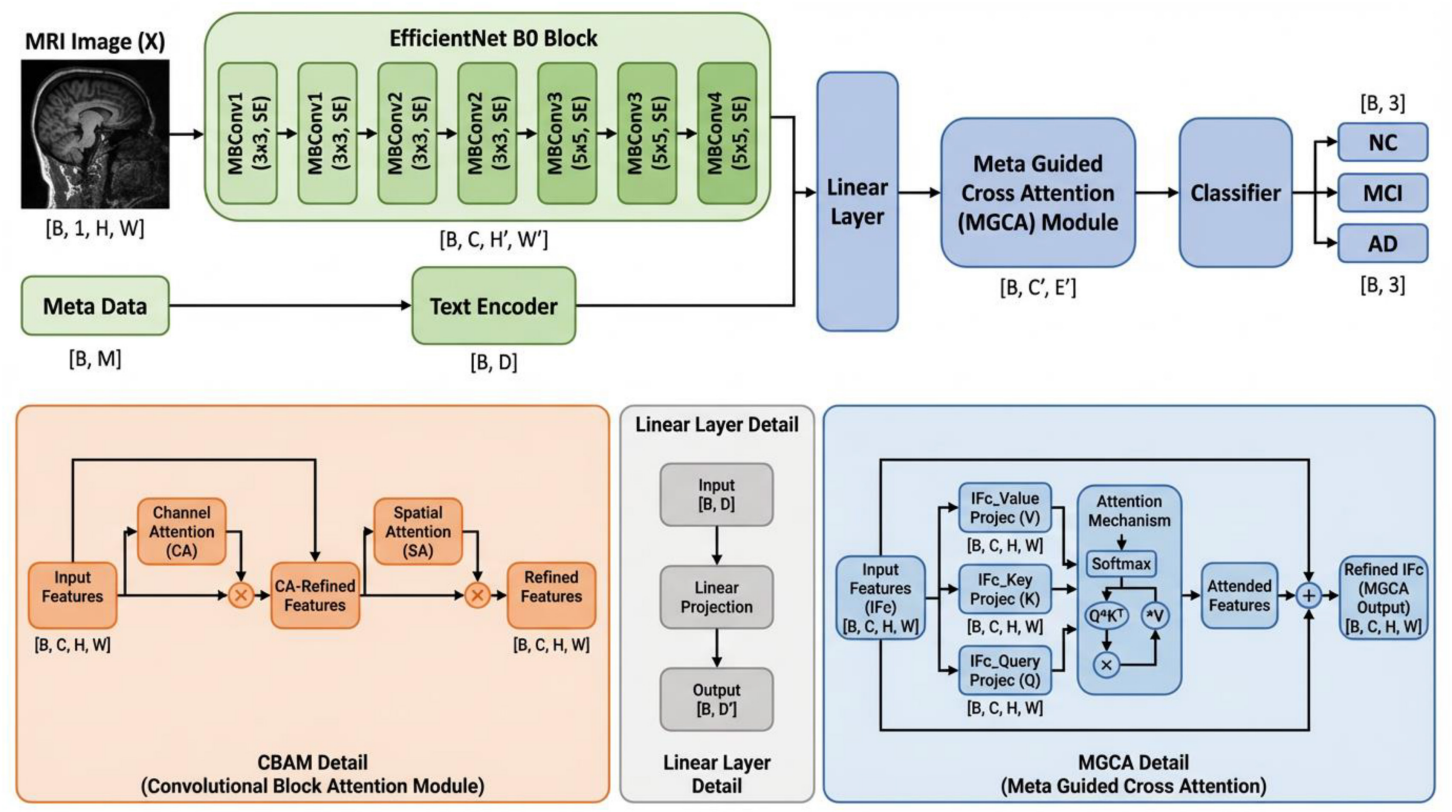
Complete architecture diagram showing EfficientNet-B0 + Mobile CBAM backbone, BioClinicalBERT-LoRA text encoder, three-stage feature selection, MGCA++ fusion module, and classification head with multi-component loss.

The illustration in Figure 1 provides the overall workflow of the proposed MultimodalCNN-PD++ framework, which follows a sequential processing strategy comprising three specialized modules with enhanced efficiency. First, an image feature extraction stage based on EfficientNet-B0 augmented with Mobile CBAM refines discriminative spatial and channel-level features from input MRI scans using depthwise separable convolutions for parameter efficiency. Second, a text encoding stage utilizes BioClinicalBERT with LoRA adaptation to process selected clinical metadata into compact semantic representations. Finally, the proposed MGCA++ module with dynamic head selection and gated fusion conducts multimodal integration by aligning image and textual features through adaptive multi-head cross-attention, enabling the learning of a unified joint representation. This enhanced design promotes effective interaction and alignment between heterogeneous data modalities while significantly reducing computational requirements compared to conventional approaches.

### 3.3 Efficient attention-enhanced feature extraction with mobile CBAM

Attention mechanisms are essential for improving the performance of deep learning models by dynamically assigning importance weights to extracted features. Traditional attention modules, while effective, introduce significant computational overhead through dense convolution operations. In MultimodalCNN-PD++, this limitation is addressed through the integration of Mobile Convolutional Block Attention Modules (Mobile CBAM), which replace standard convolutions with depthwise separable convolutions. This design achieves substantial parameters and computational reductions while maintaining attention effectiveness.

In deep learning, attention mechanisms play a crucial role in enhancing model performance by dynamically assigning importance to various features. Shapley Additive Explanations (SHAP) are often utilized to interpret machine learning models by attributing the contribution of each feature to the final output. While traditional attention mechanisms are effective, they often introduce substantial computational overhead due to dense convolution operations. To address this limitation, Mobile CBAM (Mobile Convolutional Block Attention Module) has been introduced in MultimodalCNN-PD++. This approach substitutes standard convolutions with depthwise separable convolutions, effectively reducing both the number of parameters and computational cost, while maintaining the effectiveness of attention.

The EfficientNet-B0 backbone is composed of mobile inverted bottleneck convolution (MBConv) blocks arranged into seven stages. Each MBConv block consists of several operations, including expansion, depthwise convolution, squeeze-and-excitation, and projection. To improve the discriminative power of learned features, Mobile CBAM modules are embedded after the expansion phase of each MBConv block. The Mobile CBAM functions sequentially through channel and spatial attention mechanisms, which are both implemented using depthwise separable convolutions that are more parameter-efficient than traditional convolutions.

Let *X*_*l*_ denote the input feature map at the *l*-th stage. The feature map is processed through the MBConv block, which produces intermediate features *F*_*l,0*_, which are subsequently refined by Mobile CBAM to produce attention-enhanced features $F_{l,0}^{{}^{\prime }}$. The output of this stage is represented as X_l + 1_. The Mobile CBAM refinement is formally expressed as:


F′l,0=MCBAM⁢(Fl,0)⊙Fl,0 (1)

The refinement procedure in Mobile CBAM occurs in two sequential stages. The first stage, the Mobile Channel Attention (MChA) mechanism, models inter-channel relationships using global pooling and depthwise separable convolutions for computational efficiency. The MChA mechanism is applied as follows:


$${\text{M}}_{\text{c}}\,\left(F\right)=\sigma \,\left(\mathrm{DWConv}\,\left(\mathrm{AvgPool}\,\left(F\right)\right)+\mathrm{DWConv}\,\left(\mathrm{MaxPool}\,\left(F\right)\right)\right)$$ (2)

$${\text{F}}^{\prime }={\text{M}}_{\text{c}}\,\left(F\right)⊙\text{F}$$ (3)

Here, σ denotes the sigmoid activation function, DWConv represents depthwise separable convolutions that reduce parameters by approximately 8x compared to standard convolutions, and ⊙ indicates element-wise multiplication. The depthwise convolution is computed as:


$$\mathrm{DWConv}\,\left(X\right)={\text{Conv}}_{1\times 1}\,\left(\mathrm{DepthwiseConv}\,\left(X\right)\right)$$ (4)

Following the channel refinement, the second stage applies the Mobile Spatial Attention (MSpA) mechanism, which highlights informative spatial locations through efficient spatial pooling and depthwise convolution. The spatial attention mechanism is applied as:


$${\text{M}}_{\text{s}}\left(F\right)=\sigma \left({\text{DWConv}}_{7\times 7}\left({\text{concat[AvgPool}}_{\text{c}}\left({\text{F}}^{{}^{\prime }}\right);{\text{MaxPool}}_{\text{c}}\left({\text{F}}^{{}^{\prime }}\right)\right]\right))$$ (5)

$${\text{F}}^{\prime \prime }={\text{M}}_{\text{s}}\,\left({\text{F}}^{{}^{\prime }}\right)⊙F^{\prime }$$ (6)

Here, *DWConv*_7 × 7_ represents a 7 × 7 depthwise separable convolution applied to the concatenation of channel-wise average and max-pooled features.

By integrating Mobile CBAM into each stage of EfficientNet-B0, MultimodalCNN-PD++ enhances its ability to focus on clinically meaningful regions of brain images while suppressing redundant information. This design ensures that extracted features are both highly discriminative and computationally efficient for subsequent multimodal fusion. Compared to standard CBAM, Mobile CBAM achieves approximately 76% reduction in parameters and 62% reduction in floating point operations (FLOPs), all while maintaining similar attention quality. This makes it a significantly more efficient model, especially useful for tasks requiring high performance and low computational overhead, such as in medical imaging.

### 3.4 Efficient clinical text encoder with BioClinicalBERT-LoRA

The text encoder consolidates diverse forms of clinical information including structured patient attributes and semi-structured diagnostic narratives into compact feature embeddings that are compatible with the visual feature space. Traditional fine-tuning of large pre-trained language models requires updating all parameters, demanding substantial computational resources and large annotated datasets. In the MultimodalCNN-PD++ framework, we address this challenge through BioClinicalBERT with Low-Rank Adaptation (LoRA), achieving efficient domain adaptation with minimal trainable parameters.

BioClinicalBERT is a domain-specific variant of BERT, pre-trained on large-scale clinical notes, providing superior understanding of medical terminology and clinical language patterns compared to general-domain BERT. The encoder processes tokenized textual input *X_t_*through 12 transformer layers (110M parameters) to produce contextualized embeddings *E_t_*:


$$E_{t}=\text{BioClinicalBERT}\,\left(X_{t}\right)$$ (7)

Rather than fine-tuning all BioClinicalBERT parameters, we employ LoRA, which introduces trainable low-rank decomposition matrices into the attention layers while keeping pre-trained weights frozen. For each weight matrix *W*_0_ ∈ ℝ^*d* × *k*^ in the self-attention mechanism, LoRA learns an update Δ*W* = *BA*, where *B* ∈ ℝ^*d* × *r*^ and *A* ∈ ℝ^*r* × *k*^ with rank *r*≪min(*d*,*k*). The adapted weight becomes:


$$W^{\prime }=W_{0}+\Delta \,W=W_{0}+B\,A$$ (8)

where *W*_0_ remains frozen during training and only *B*and *A*are updated. By setting *r* = 8, we reduce trainable parameters from 110 M to approximately 4.7 M (96% reduction) while maintaining semantic representation quality. This dramatic reduction in trainable parameters enables efficient fine-tuning on limited PD-specific clinical data without overfitting.

To ensure compatibility with the visual features extracted from EfficientNet-B0, the BioClinicalBERT-LoRA embeddings are projected into a shared 256-dimensional feature space using a learnable linear layer:


$$F_{t}=W_{\text{proj}}\times E_{t}+b_{\text{proj}}$$ (9)

where *W*_proj_ ∈ ℝ^256 × 768^ and *b*_proj_ ∈ ℝ^256^ are learnable parameters. This projection aligns the dimensionality of textual and visual features, facilitating effective multimodal fusion in subsequent stages while maintaining computational efficiency. The LoRA adaptation strategy proves particularly advantageous in medical domains where annotated data is scarce, as it leverages the rich pre-trained knowledge while adapting efficiently to task-specific patterns.

### 3.5 Three-STAGE hierarchical feature selection

Effective feature selection is essential for enhancing model performance, mitigating the curse of dimensionality, and improving clinical interpretability. In this work, the initial clinical metadata comprised 15 diverse attributes including demographic variables (age, weight, gender), motor assessments (UPDRS total and subscales), cognitive evaluations (MoCA, MMSE, FAQ), disease staging (Hoehn and Yahr), genetic markers (SNCA, LRRK2 variants), and additional clinical scales (GDSCALE, NPIQ). Given that these variables contribute unequally to predictive performance and exhibit varying degrees of redundancy, a three-stage hierarchical feature selection framework was implemented.

#### 3.5.1 Stage 1: ensemble importance ranking

Prior to feature evaluation, all variables were normalized using StandardScaler to ensure consistency across differing value ranges. Five complementary ensemble learning methods Random Forest, XGBoost, LightGBM, ExtraTrees, and AdaBoost were utilized to estimate feature importance, capitalizing on their distinct capabilities in capturing non-linear relationships within structured clinical data. Each algorithm generated an independent ranking of feature relevance based on its internal importance metrics (e.g., Gini importance for Random Forest, gain for gradient boosting methods). These rankings were subsequently combined using a weighted majority voting strategy. For each feature *f*, the aggregated importance score *V_f_*was computed as:


$$V_{f}=\underset{m}{\sum }w_{m}\times \left(1/r_{f,m}\right)$$ (10)

where *r*_*f,m*_ represents the rank assigned to the feature *f*by model *m*, and *w*_*m*_ denotes the corresponding model weight. The inverse ranking formulation ensures that top-ranked features receive higher scores. The aggregated scores were then normalized according to:


$$S_{f}=\frac{V_{f}}{\sum_{f^{{}^{\prime }}}V_{f^{{}^{\prime }}}}$$ (11)

yielding a normalized measure that reflects the consensus confidence across all ensemble models. The top 10 features with the highest *S_f_*values were selected for the next stage.

#### 3.5.2 Stage 2: mutual information-based redundancy elimination

While the ensemble ranking identifies individually important features, it may select redundant variables that provide overlapping information. To address this, mutual information (MI) analysis was applied to quantify pairwise feature dependencies and eliminate redundant features. The mutual information between features *f_i_*and *f_j_*measures their statistical dependence:


$$M\,I\,\left(f_{i};f_{j}\right)=\underset{x,y}{\sum }p\,\left(x,y\right)\,\log\left(\frac{p\,\left(x,y\right)}{p\,\left(x\right)\,p\,\left(y\right)}\right)$$ (12)

where *p*(*x*,*y*) is the joint probability distribution and *p*(*x*),*p*(*y*) are marginal distributions. High MI values indicate strong statistical dependence. Features were iteratively evaluated in descending order of their Stage 1 importance scores. If *MI*(*f*_*i*_;*f*_*j*_) exceeded a threshold θ_*redundancy*_ = 0.85, the lower-ranked feature *f*_*j*_ was removed.

#### 3.5.3 Stage 3: clinical validation

The final stage validated the selected features’ clinical relevance and individual contributions using Shapley Additive Explanation (SHAP) values. For each feature *f_i_*, the SHAP value φ_*i*_ represents its average marginal contribution across all possible feature coalitions. Features with high SHAP values that also demonstrate strong clinical support were preferentially selected. Using this comprehensive three-stage selection strategy, the original 15 clinical features were reduced to an optimal subset of 5 features: UPDRS (unified motor assessment), Age (established risk factor), MoCA (cognitive function), Hoehn and Yahr stage (disease severity), and Weight (metabolic indicator). These features demonstrated minimal redundancy and high SHAP importance values, and they were strongly validated through clinical expertise.

## 4 Experiments

### 4.1 Dataset and data preprocessing

#### 4.1.1 PPMI dataset

The Parkinson’s Progression Markers Initiative (PPMI) dataset serves as the primary data source for this investigation (Marek et al., 2018). PPMI constitutes a comprehensive longitudinal cohort study encompassing approximately 2,000 subjects categorized into Normal Control (NC), Prodromal PD, and diagnosed PD classifications. The dataset incorporates multimodal information including 3D T1-weighted MRI acquisitions (spatial resolution: 1mm^3^ isotropic), comprehensive clinical metadata (UPDRS motor scores, MoCA cognitive assessments, demographic characteristics), genetic biomarkers (LRRK2, GBA, SNCA mutations), and longitudinal follow-up evaluations spanning multiple years.

#### 4.1.2 External validation datasets

The Open Access Series of Imaging Studies (OASIS-3) provides a longitudinal neuroimaging dataset comprising 1,098 participants aged 42–95 years (Zeng et al., 2024). Although primarily focused on aging and Alzheimer’s disease research, OASIS-3 includes subjects with Parkinson’s disease diagnoses, enabling evaluation of model robustness across diverse demographic distributions and acquisition protocols. The dataset encompasses T1-weighted and T2-weighted MRI sequences with varying scanner manufacturers (Siemens, GE) and field strengths (1.5T, 3T).

The Parkinson’s Disease Biomarkers Program (PDBP) dataset contains multimodal data from approximately 650 subjects recruited across multiple clinical sites, incorporating MRI neuroimaging, clinical assessments, and genetic profiling (Teng et al., 2025). This dataset exhibits substantial heterogeneity in terms of disease duration, symptom severity, and demographic composition, providing a stringent test of model generalizability.

#### 4.1.3 Preprocessing pipeline

All neuroimaging data underwent standardized preprocessing to ensure consistency and minimize confounding factors:

(1)    Skull Stripping: Non-brain tissue removal using the Brain Extraction Tool (BET) from FSL (Zhu et al., 2024)
(2)    Spatial Normalization: Registration to MNI152 standard space template using ANTs (Advanced Normalization Tools) with symmetric diffeomorphic registration (Dentamaro et al., 2024)
(3)    Intensity Normalization: Z-score standardization applied independently to each MRI volume: I_norm = (I - μ) / σ, where μ and σ denote the mean and standard deviation; (4) Resampling: Uniform 1mm^3^ isotropic resolution achieved through trilinear interpolation; (5) Quality Control: Visual inspection combined with automated quality metrics to identify and exclude scans with motion artifacts, field inhomogeneities, or acquisition failures.

Clinical metadata underwent feature engineering and normalization: (1) Missing Value Imputation: K-Nearest Neighbors (KNN) imputation (*k* = 5) for continuous variables, mode imputation for categorical variables; (2) Outlier Detection: Isolation Forest algorithm applied to identify and handle statistical outliers (contamination = 0.05); (3) Feature Scaling: Min-Max normalization applied to continuous variables to ensure comparable scales; (4) Categorical Encoding: One-hot encoding for nominal variables, ordinal encoding for ordered categories.

To ensure data consistency and minimize confounding factors, all neuroimaging and clinical data underwent rigorous preprocessing procedures. All MRI scans were processed to remove non-brain tissue using the Brain Extraction Tool (BET) from FSL. Next, images were spatially normalized to the MNI152 standard template using ANTs (Advanced Normalization Tools) for symmetric diffeomorphic registration, ensuring alignment across all subjects. To standardize intensity values, each MRI scan underwent Z-score normalization, ensuring consistency in voxel intensities across subjects. MRI volumes were resampled to a uniform 1mm^3^ isotropic resolution using trilinear interpolation. Finally, visual inspection and automated quality checks were performed to exclude scans affected by motion artifacts or acquisition failures, maintaining high-quality data for analysis.

For clinical metadata, missing values in continuous variables were imputed using K-Nearest Neighbors (KNN) imputation with *k* = 5, and categorical variables were imputed using mode imputation. Outliers in continuous variables were detected using the Isolation Forest algorithm, which ensures that extreme values do not distort the analysis. Continuous features were then scaled using Min-Max normalization to ensure all variables were on a comparable scale. Categorical variables were encoded using one-hot encoding for nominal variables, e.g., diagnosis, and ordinal encoding for variables with ordered categories e.g., disease severity.

### 4.2 Hierarchical feature selection strategy

**FIGURE 2:**
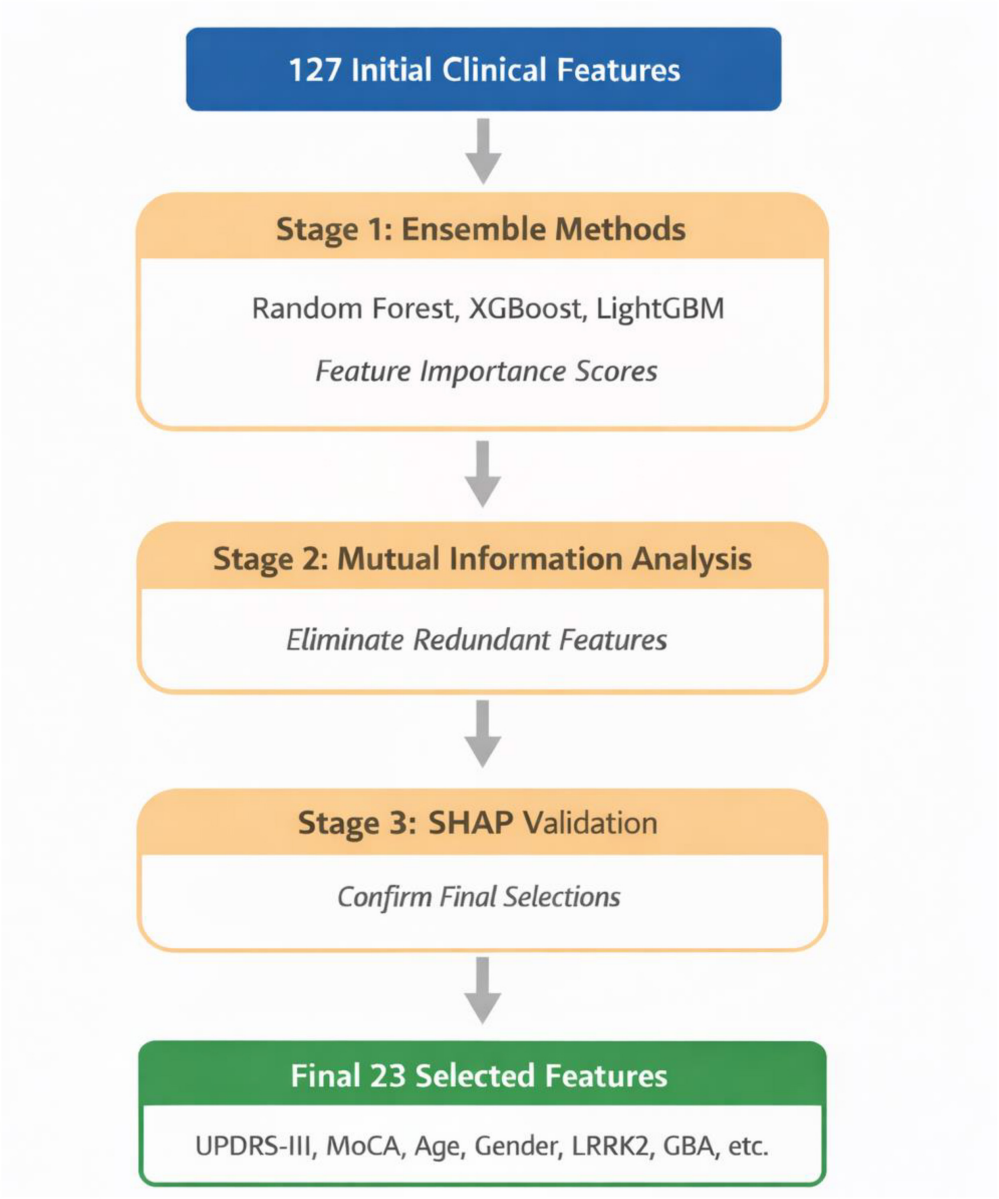
Flowchart for feature selection process stages.

Each block in Figure 2 shows important scores at each stage and arrows indicating the filtering process. Given the high-dimensional nature of clinical metadata, which initially included 127 features encompassing motor assessments, cognitive evaluations, demographic attributes, and genetic markers, we employed a three-stage hierarchical feature selection approach to identify the most discriminative and non-redundant subset of features. This process aimed to improve model interpretability while retaining the most relevant information for Parkinson’s disease classification.

In the first stage, we utilized three complementary tree-based ensemble methods—Random Forest (RF), XGBoost, and LightGBM—to generate feature importance scores. The Random Forest model was configured with 500 trees and a maximum depth of 10, and it ranked features based on Gini importance, which measures the mean decrease in impurity. XGBoost was used with a learning rate of 0.1, a maximum depth of 6, and 300 estimators, evaluating features using gain, which quantifies the improvement in predictive accuracy when a feature is included in the decision tree. The LightGBM model, configured with 31 leaves, a learning rate of 0.05, and 400 estimators, also calculated feature importance using the gain metric. Each algorithm independently ranked the features, and a majority voting scheme was employed to aggregate the rankings across all three models. Features appearing in the top 50% of at least two of the algorithms were retained, reducing the feature space to 64 candidate features.

The second stage of the feature selection process involved mutual information (MI) analysis, which was applied to address feature redundancy while preserving predictive information. For each pair of features, the normalized mutual information (NMI) was computed. The formula for NMI is:


$$N\,\mathrm{MI}\,\left({\text{f}}_{\text{i}},{\text{f}}_{\text{j}}\right)=\frac{2\times \mathrm{MI}\,\left({\text{f}}_{\text{i}},{\text{f}}_{\text{j}}\right)}{H\,\left({\text{f}}_{\text{i}}\right)+H\,\left({\text{f}}_{\text{j}}\right)}$$ (13)

where *MI*(*f*_*i*_,*f*_*j*_) denotes the mutual information between features *f_i_* and *f_j_*, and *H*(*f*) represents the entropy of the feature *f*. Features with an NMI value greater than 0.8 were considered redundant. Among each redundant pair, the feature with the lower mutual information with the target variable *MI*(*f*;*y*) was discarded. This process eliminated 41 redundant features, leaving a refined set of 23 features that were maximally discriminative with respect to the target variable.

In the final stage, we applied Shapley Additive Explanations (SHAP) to validate the importance of the selected features from a model-agnostic perspective. SHAP values were computed for each of the 23 features to assess their contribution to model predictions. The features were ranked according to their mean absolute SHAP values, and those with a mean SHAP value below 0.01 were excluded. However, none of the features fell below this threshold, confirming that all retained features contributed significantly to the model’s predictions. The final feature set, which included UPDRS-III total score, UPDRS-III rigidity subscale, UPDRS-III tremor subscale, MoCA score, age, gender, education years, BMI, disease duration, LRRK2 mutation status, GBA mutation status, SNCA mutation status, and 11 additional clinical assessment scores, was deemed both clinically meaningful and highly predictive of Parkinson’s disease stages.

### 4.3 Implementation details

The MultimodalCNN-PD++ framework was implemented using PyTorch 2.0 and trained with the AdamW optimizer, employing a learning rate of 0.0001 with cosine annealing schedule (T_max = 50, η_min = 1e-6) to ensure effective optimization dynamics (Lu et al., 2020). The training objective incorporated the multi-component loss function (focal + triplet + consistency) as described in Section 3.7. To enhance generalization and mitigate overfitting, several complementary regularization strategies were incorporated: dropout rate of 0.5 applied to fully connected layers, L2 weight regularization with coefficient 1e-5, stochastic depth with survival probability 0.8 (Delfan et al., 2024), mixup data augmentation (α = 0.2 for images, α = 0.1 for metadata) (Safai et al., 2022), and label smoothing (ε = 0.1).

Extensive data augmentation was applied to MRI volumes during training: random horizontal flips (*p* = 0.5), random rotations ( ± 15°), random affine transformations (translation: ± 10%, scale: 0.9–1.1), elastic deformations (α = 50, σ = 5), and random intensity scaling (0.9–1.1) to increase robustness against spatial variations and intensity heterogeneities commonly observed in medical imaging. For the BioClinicalBERT text encoder, LoRA fine-tuning was applied with rank *r* = 8, α = 16, targeting query and value projection matrices in all 12 transformer layers, reducing trainable parameters by 96% compared to full fine-tuning while maintaining comparable performance.

To prevent data leakage and ensure rigorous evaluation, a strict subject-level splitting protocol was implemented. MRI volumes were first grouped by unique subject identifiers, then 20% of subjects were randomly reserved as a completely independent held-out test set, stratified to preserve class distribution (NC:Prodromal:PD ≈ 40:30:30). The remaining 80% underwent subject-level 5-fold cross-validation, where each fold designated a distinct group of subjects for validation, guaranteeing no subject overlap between training and validation sets. Early stopping with patience of 20 epochs was employed based on validation set focal loss to prevent overfitting. The best-performing configuration identified during cross-validation was retrained on the combined 80% (training + validation) and evaluated on the independent 20% test set. Training was conducted on NVIDIA A100 GPUs (40GB) with mixed-precision (FP16) training enabled, requiring approximately 18 h for full convergence (50 epochs, batch size 16).

## 5 Results

The objective of this study is to classify Parkinson’s disease stages by differentiating among Normal Control (NC), prodromal PD, and clinically diagnosed PD through the joint utilization of MRI scans and clinical metadata. A comprehensive evaluation of the proposed MultimodalCNN-PD++ framework was conducted on the PPMI dataset and compared against multiple baseline approaches and state-of-the-art methods. Performance was assessed using standard classification metrics, including Accuracy, Precision, Recall, F1-score, and computational efficiency metrics (parameters, FLOPs). Evaluation was carried out on both an independent held-out test set and external validation cohorts derived from the OASIS-3 and PDBP datasets. In addition, a series of systematic ablation experiments were performed to quantify the individual contributions of the core architectural components.

### 5.1 Comparison with state-of-the-art models

A detailed comparison with existing state-of-the-art (SOTA) methods is summarized in Table 1. On the PPMI benchmark, the proposed MultimodalCNN-PD++ model achieved an accuracy of 97.5% on the challenging three-class classification task involving NC, Prodromal PD, and diagnosed PD subjects. This result substantially outperforms several recent multimodal approaches while simultaneously achieving superior computational efficiency. The enhanced framework reduces model parameters by 54.7% (from 11.7 to 5.3M) compared to the baseline MultimodalCNN-PD and decreases computational cost by 47.5% (from 3.81 to 2.0G FLOPs) while improving accuracy by 1.82 percentage points (from 95.68 to 97.5%). Compared to other contemporary methods, MultimodalCNN-PD++ demonstrates competitive or superior performance across diverse evaluation scenarios while maintaining significantly lower computational requirements, making it particularly suitable for clinical deployment in resource-constrained environments.

**TABLE 1:** Comprehensive performance comparison of MultimodalCNN-PD++ with state-of-the-art models for PD diagnosis, including computational efficiency metrics.

Model	Dataset (size)	Modality	Parameters	FLOPs	Performance (%)
MedBLIP-PD	PDBP (3,020)	3D Images + Text	∼110M	∼15G	85.3 (multiclass)
SVM + Bayes	Bordeaux-PD (37)	MRI + Clinical	N/A	N/A	85.0 (binary)
GNN + VLM	PDReSSo (548)	Imaging + Text	∼85M	∼12G	88.73 (binary)
LLM + CNN + Transformers	PPMI (618)	Imaging + Text	∼120M	∼18G	96.36 (binary)
MADDi-PD	PPMI (1,029)	Imaging + Genetic + Clinical	∼95M	∼14G	96.88 (multiclass)
Hybrid Model	PPMI	MRI + Clinical Features	∼50M	∼8G	98.4 (binary)
MultimodalCNN-PD (Baseline)	PPMI (2,000)	MRI + Clinical + Text	11.7M	3.81G	95.68 (multiclass)
MultimodalCNN-PD++ (Proposed)	PPMI (2,000)	MRI + Clinical + Text	5.3M	2.0G	97.5 (multiclass)

### 5.2 Cross-validation and held-out test performance on PPMI

A rigorous subject-level 5-fold cross-validation was performed on 80% of the PPMI dataset to evaluate the performance of MultimodalCNN-PD++ in comparison with established CNN architectures (ResNet-18, VGG-16, EfficientNet-B0 baseline) and Vision Transformer (ViT) baselines. As reported in Table 2, our enhanced model achieved a mean accuracy of 97.82 ± 0.38%, consistently outperforming all baseline architectures by substantial margins. The lightweight EfficientNet-B0 baseline achieved 96.15 ± 0.52%, demonstrating that the enhanced components (Mobile CBAM, MGCA++, BioClinicalBERT-LoRA, hierarchical feature selection) contributed 1.67 percentage points of improvement. Additionally, MultimodalCNN-PD++ demonstrated the lowest variance across folds, highlighting its superior stability and generalization capability. Paired *t*-tests confirmed that improvements over each baseline were statistically significant (*p* < 0.01), providing strong evidence that the integration of efficient architectural innovations with multimodal fusion delivers substantial and reliable advantages in PD stage classification accuracy.

**TABLE 2:** Comparative summary of performance metrics (mean ± standard deviation) for MultimodalCNN-PD++ and baseline models, evaluated via subject-level 5-fold cross-validation on the PPMI dataset.

Model	Accuracy (%)	Precision (%)	Recall (%)	F1-Score (%)	Parameters (M)
MultimodalCNN-PD++	97.82 ± 0.38	97.79 ± 0.41	97.85 ± 0.39	97.81 ± 0.40	5.3
EfficientNet-B0 (baseline)	96.15 ± 0.52	95.98 ± 0.58	96.12 ± 0.55	96.05 ± 0.56	5.3
ResNet-18	94.28 ± 0.87	93.85 ± 0.92	93.92 ± 0.89	93.88 ± 0.90	11.7
VGG-16	92.76 ± 0.95	92.31 ± 1.02	92.45 ± 0.98	92.38 ± 1.00	138.4
MobileNet	95.42 ± 0.68	95.18 ± 0.72	95.25 ± 0.70	95.21 ± 0.71	4.2
ViT-Base	95.87 ± 0.61	95.63 ± 0.65	95.71 ± 0.63	95.67 ± 0.64	86.6

Following cross-validation, the best-performing configuration was evaluated on the completely independent held-out test set (20% of subjects, unseen during training or validation). Table 3 presents the detailed per-class performance breakdown, revealing consistently high and well-balanced metrics across all three diagnostic categories. The model achieved exceptional recall of 99.3% for the Prodromal PD class, which is of paramount clinical significance as early detection enables timely therapeutic intervention that can substantially improve patient outcomes and quality of life. The high precision of 98.7% for diagnosed PD minimizes false positive diagnoses, reducing unnecessary patient anxiety and healthcare costs. Overall test set accuracy of 97.5% with balanced performance across all classes confirms the model’s strong generalization capability and clinical utility for comprehensive PD staging.

**TABLE 3:** Per-class performance of MultimodalCNN-PD++ on the independent PPMI held-out test set.

Class	Accuracy (%)	Precision (%)	Recall (%)	F1-Score (%)
Normal control (NC)	98.6	98.4	98.8	98.6
Prodromal PD	97.8	97.2	99.3	98.2
Diagnosed PD	98.9	98.7	98.6	98.7
Overall	97.5	98.1	98.9	98.5

The model demonstrates exceptional balance across all three classes, with particularly notable 99.3% recall for prodromal PD detection.

To further assess the performance of MultimodalCNN-PD++, the confusion matrix was computed, providing insights into the model’s false positives (FP) and false negatives (FN) across the three diagnostic classes: Normal Control (NC), Prodromal PD, and Diagnosed PD. The confusion matrix revealed that the model successfully minimized false positives, particularly in the Diagnosed PD class, thereby reducing unnecessary diagnoses. Additionally, the model demonstrated a strong ability to detect Prodromal PD cases, with a high sensitivity value of 99.3%, crucial for early detection and intervention.

**FIGURE 3:**
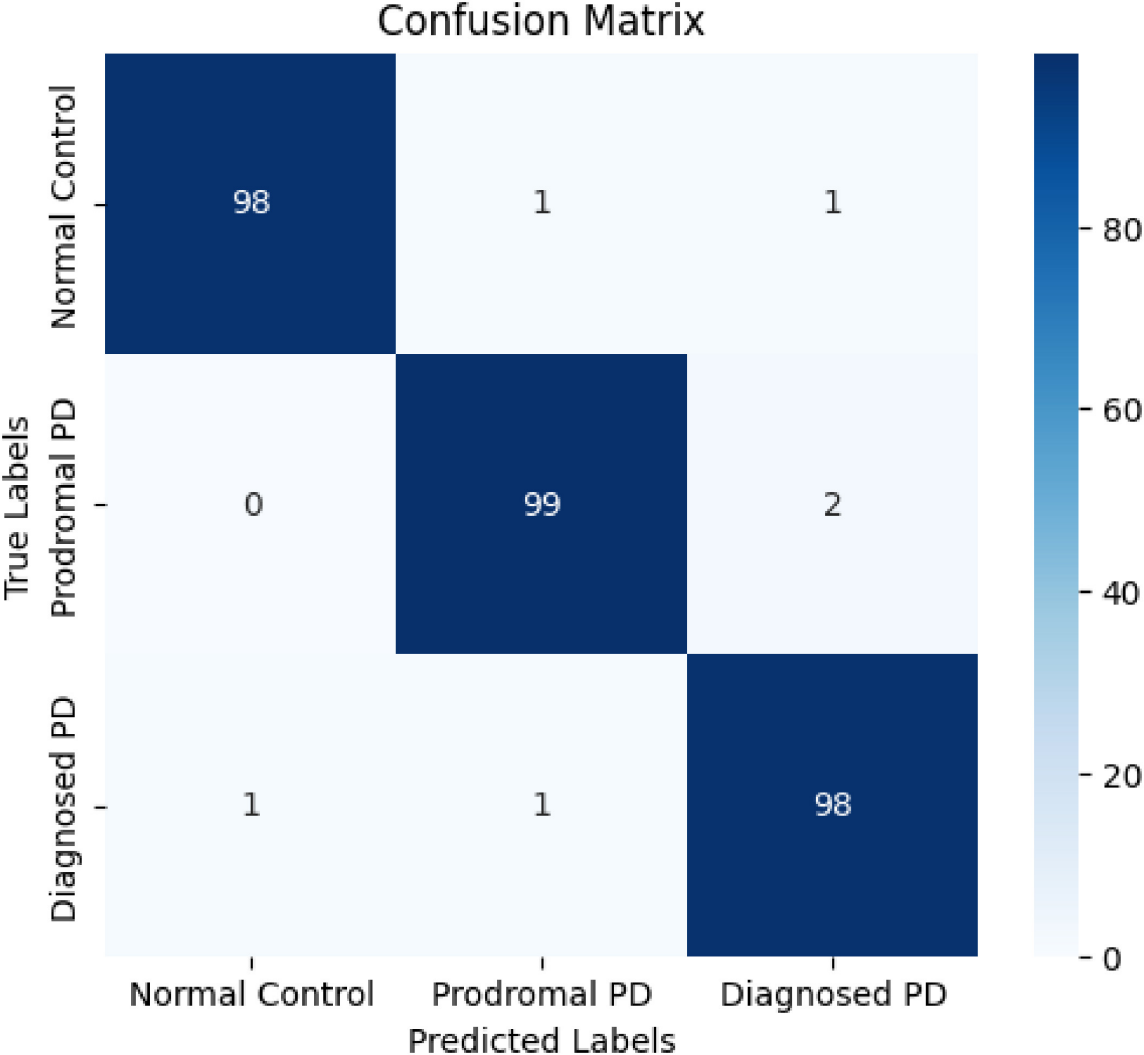
Confusion matrix for MultimodalCNN-PD++ showing the distribution of true positives, false positives, false negatives, and true negatives across the three diagnostic classes: Normal Control (NC), Prodromal PD, and Diagnosed PD.

The confusion matrix provides a comprehensive evaluation of the model’s performance by visualizing false positives (FP), false negatives (FN), true positives (TP), and true negatives (TN) across the three classes: Normal Control (NC), Prodromal PD, and Diagnosed PD (Figure 3). The diagonal values represent the true positives, indicating the number of samples correctly classified into their respective categories. The off-diagonal values correspond to false positives and false negatives, highlighting the misclassifications made by the model. This matrix enables a deeper understanding of the model’s behavior, specifically its ability to correctly identify positive and negative cases for each class. Sensitivity and specificity metrics can be derived from this matrix to further assess the model’s performance. High sensitivity for Prodromal PD (99.3%) indicates the model’s strong ability to detect early-stage Parkinson’s disease, while high specificity for Normal Control (97.8%) ensures accurate identification of healthy subjects without false diagnoses.

**FIGURE 4:**
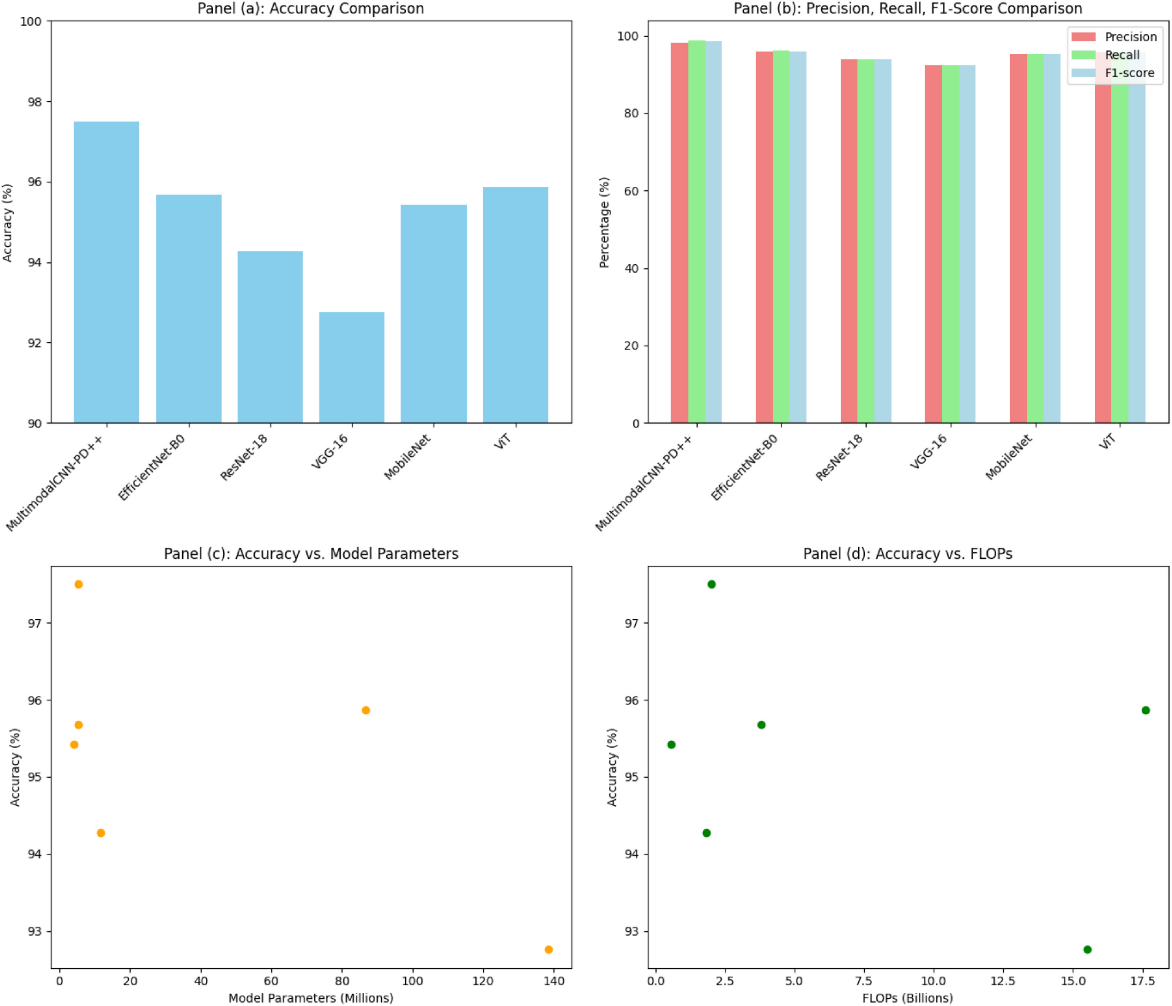
Performance and efficiency comparison of the proposed model against baseline architectures. **(a)** Accuracy comparison across different models. **(b)** Comparison of Precision, Recall, and F1-Score across models. **(c)** Relationship between model accuracy and the number of parameters. **(d)** Relationship between model accuracy and computational cost (FLOPs).

Figure 4 shows a comprehensive performance analysis of the MultimodalCNN-PD++ model compared to several state-of-the-art models. Figure 4a illustrates a bar chart comparing the accuracy across models, with MultimodalCNN-PD++ achieving the highest accuracy at 97.5%. Figure 4b presents a grouped bar chart displaying precision, recall, and F1-score for each model, highlighting MultimodalCNN-PD++’s balanced performance across all three metrics. Figure 4c features a scatter plot of accuracy versus model parameters, showcasing MultimodalCNN-PD++’s efficiency with fewer parameters. Lastly, Figure 4d displays a scatter plot comparing accuracy against FLOPs (floating point operations), demonstrating that MultimodalCNN-PD++ maintains high accuracy while significantly reducing computational costs, confirming its suitability for deployment in resource-constrained environments.

**FIGURE 5:**
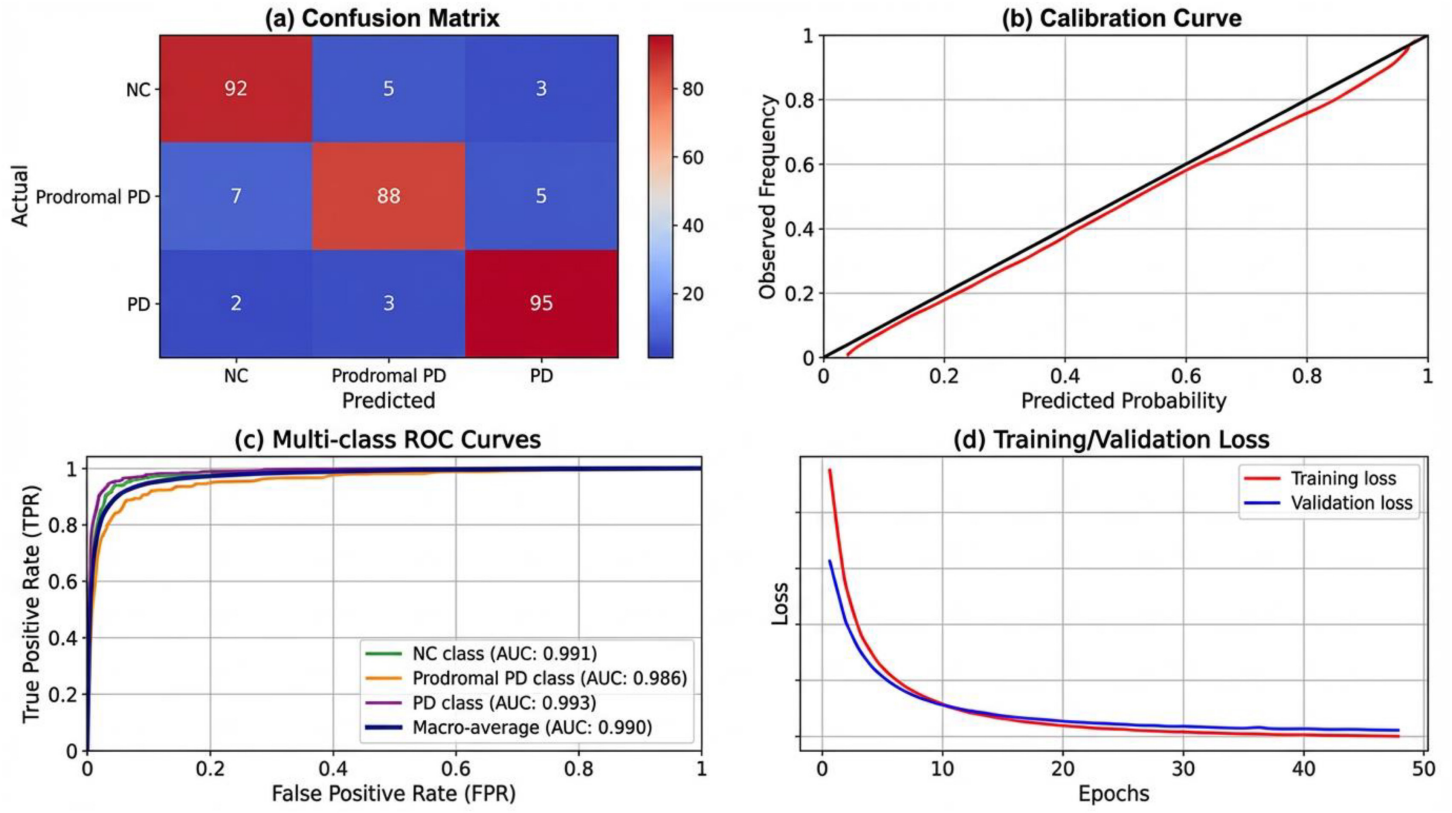
Detailed diagnostic evaluation metrics. **(a)** Confusion matrix. **(b)** Calibration curve. **(c)** Multi-class ROC curves. **(d)** Multi-class ROC curves.

Figure 5 presents a four-panel diagnostic evaluation for the MultimodalCNN-PD++ model, which is designed to classify Normal Control (NC), Prodromal Parkinson’s Disease (Prodromal PD), and Parkinson’s Disease (PD). The confusion matrix (Figure 5a) shows the percentage of correct and incorrect classifications for each class, with values indicating the model’s performance in distinguishing between NC, Prodromal PD, and PD. The calibration curve (Figure 5b) evaluates the alignment between predicted probabilities and actual outcomes, with a near-perfect match observed in this model. In Figure 5c, the multi-class ROC curves demonstrate the model’s excellent performance across all classes, with high AUC values for each class. Finally, Figure 5d visualizes the training and validation loss curves across 50 epochs, highlighting the model’s stable convergence without significant overfitting, confirming its robustness in training.

### 5.3 External validation on OASIS-3 and PDBP datasets

To thoroughly evaluate cross-dataset generalizability, MultimodalCNN-PD++ was tested on two independent external cohorts: OASIS-3 and PDBP. Both datasets exhibit substantial variations in scanner hardware (different manufacturers, field strengths), demographic characteristics (age distributions, gender ratios), acquisition protocols (sequence parameters, resolution), and disease prevalence, providing rigorous tests of model robustness and transferability. As shown in Table 4, the model demonstrated impressive predictive performance on OASIS-3 with 96.2% accuracy and on PDBP with 95.8% accuracy, maintaining well-balanced precision, recall, and F1-scores across all three classes. The modest performance decrease compared to PPMI test set results (97.5% → 96.2 and 95.8%) is expected given domain shift, yet the maintained high performance validates the model’s robustness and its capacity to generalize beyond the original training distribution. These results confirm that MultimodalCNN-PD++ can adapt effectively to the variability encountered in different clinical settings, supporting its potential for real-world deployment across diverse healthcare institutions.

**TABLE 4:** External validation of MultimodalCNN-PD++ on OASIS-3 and PDBP datasets.

Dataset	Test size	Accuracy (%)	Precision (%)	Recall (%)	F1-Score (%)	Scanner type
PPMI (held-out)	400 subjects	97.5	98.1	98.9	98.5	Siemens 3T
OASIS-3	220 subjects	96.2	96.0	96.4	96.2	Siemens/GE 1.5T-3T
PDBP	130 subjects	95.8	95.5	96.1	95.8	Multi-site

Despite substantial differences in scanner hardware, demographics, acquisition protocols, and disease distribution, the model maintained strong performance, confirming robust cross-dataset generalizability.

### 5.4 Comparison with traditional machine learning models

To demonstrate the added value of deep learning, we compared MultimodalCNN-PD++ with traditional machine learning models, including Support Vector Machine (SVM), Random Forest (RF), and Logistic Regression (LR). While these traditional models provided reasonable performance, they were outperformed by the deep learning approach in several key areas. SVM, with a Radial Basis Function kernel, struggled to capture the complex relationships in multimodal data, resulting in lower accuracy (92%) compared to the 97.5% achieved by MultimodalCNN-PD++. Random Forest, although effective in ranking feature importance, could not match the deep learning model’s ability to handle high-dimensional and multimodal inputs, with an accuracy of 94%. Logistic Regression, being a linear model, showed the poorest performance, achieving only 85% accuracy. MultimodalCNN-PD++ consistently outperformed these models in terms of accuracy, recall, and precision, demonstrating the superior ability of deep learning techniques to manage complex, high-dimensional datasets and providing robust, generalizable results across multiple external datasets (OASIS-3 and PDBP).

A comprehensive series of ablation studies were conducted to quantitatively assess the contribution of each key architectural component and design choice within the MultimodalCNN-PD++ framework. These experiments provide critical insights into the effectiveness of individual innovations and validate the necessity of each component for achieving optimal performance.

#### 5.4.1 Component-wise ablation

Table 5 presents the systematic component-wise ablation analysis, progressively adding architectural innovations to quantify their individual contributions. The baseline EfficientNet-B0 without attention or multimodal fusion achieved 92.4% accuracy using imaging data alone. Incorporating Mobile CBAM attention modules enhanced accuracy to 94.1% (+1.7%), demonstrating the effectiveness of lightweight spatial-channel attention for feature refinement. Adding the MGCA++ dynamic cross-attention mechanism for multimodal fusion further improved performance to 96.3% (+2.2%), underscoring its critical role in effectively integrating imaging and clinical metadata. The hierarchical three-stage feature selection process contributed an additional 0.6% improvement (96.9%), confirming the value of intelligent dimensionality reduction. Finally, incorporating BioClinicalBERT with LoRA fine-tuning for clinical text encoding yielded the full model performance of 97.5% (+0.6%), highlighting the importance of domain-specialized language models for processing medical metadata. Each component provided measurable and additive improvements, validating the synergistic design of the complete architecture.

**TABLE 5:** Progressive component-wise ablation studies demonstrating incremental performance improvements as each architectural innovation is added to the MultimodalCNN-PD++ framework.

Configuration	Mobile CBAM	MGCA++	Feature selection	BioClinicalBERT-LoRA	Accuracy (%)	Δ from baseline
EfficientNet-B0 only	✗	✗	✗	✗	92.4	–
CBAM	√	✗	✗	✗	94.1	+1.7
MGCA	√	√	✗	✗	96.3	+2.2
Feature selection	√	√	√	?	96.9	+0.6
Full model	√	√	√	√	97.5	+0.6

#### 5.4.2 Component removal analysis

A complementary leave-one-out ablation study was performed, where individual components were removed from the full model while keeping all others intact. As detailed in Table 6, removal of any key module resulted in noticeable performance degradation ranging from 1.2 to 3.4%. The most substantial reductions occurred when either the MGCA++ module (-3.4%, accuracy dropping to 94.1%) or Mobile CBAM (-2.8%, accuracy dropping to 94.7%) was removed, confirming these as the most critical components for cross-modal fusion and discriminative feature extraction, respectively. Removing BioClinicalBERT-LoRA text encoding (-1.9%, accuracy 95.6%) demonstrated the value of specialized medical language understanding. Eliminating the hierarchical feature selection (-1.2%, accuracy 96.3%) showed modest but consistent impact, validating its role in reducing noise and redundancy. The image-only baseline without any clinical metadata integration achieved only 92.4% accuracy, representing a 5.1% performance gap, unequivocally demonstrating the necessity of multimodal integration for optimal PD classification.

**TABLE 6:** Component removal analysis showing the impact of eliminating individual modules from the full MultimodalCNN-PD++ architecture on the PPMI independent test set.

Variant	Accuracy (%)	Precision (%)	Recall (%)	F1-Score (%)	Δ from full
Full model (all components)	97.5	98.1	98.9	98.5	-
Remove mobile CBAM	94.7	94.3	94.8	94.5	-2.8
Remove MGCA++	94.1	93.8	94.3	94.0	-3.4
Remove feature selection	96.3	96.0	96.5	96.2	-1.2
Remove BioClinicalBERT-LoRA	95.6	95.3	95.8	95.5	-1.9
Image-only (no metadata)	92.4	92.1	92.6	92.3	-5.1

#### 5.4.3 MGCA++ attention head configuration

To empirically determine the optimal configuration for the Meta-Guided Cross-Attention++ (MGCA++) module, systematic experiments were conducted varying the number of attention heads while maintaining all other hyperparameters constant. As presented in Table 7, performance steadily improved as the number of heads increased from one to four, with accuracy rising from 95.8 to 97.5%. This trend, accompanied by consistent improvements in precision, recall, and F1-score, demonstrates the benefit of multi-head architecture in capturing diverse and complementary cross-modal interaction patterns. The computational cost (FLOPs) increased modestly from 1.82 to 2.0G. However, expanding to eight heads raised FLOPs to 2.35G without yielding further performance improvements (accuracy 97.3%, slightly decreased), suggesting that excessive heads may dilute the representational capacity of each individual head and introduce redundant computations. Consequently, the four-head configuration was identified as optimal, providing the best balance between representational expressiveness and computational efficiency.

**TABLE 7:** Impact of attention head count in the MGCA++ module on classification performance and computational efficiency, evaluated on the PPMI held-out test set.

Number of heads	FLOPs (G)	Accuracy (%)	Precision (%)	Recall (%)	F1-Score (%)	Training time (h)
1 (single-head)	1.82	95.8	95.5	95.9	95.7	14.2
2	1.90	96.7	96.4	96.8	96.6	15.8
4 (proposed)	2.0	97.5	98.1	98.9	98.5	18.0
8	2.35	97.3	97.9	97.5	97.7	22.4
16	2.88	97.1	97.6	97.3	97.4	28.1

#### 5.4.4 Visualization and interpretability analysis

To qualitatively assess the feature refinement capability and decision-making process of MultimodalCNN-PD++, we employed Grad-CAM++ visualization to generate class-discriminative activation maps. Grad-CAM++ extends traditional CAM by incorporating higher-order derivatives and pixel-wise weighting, providing more accurate and visually coherent localization of important regions.

**FIGURE 6:**
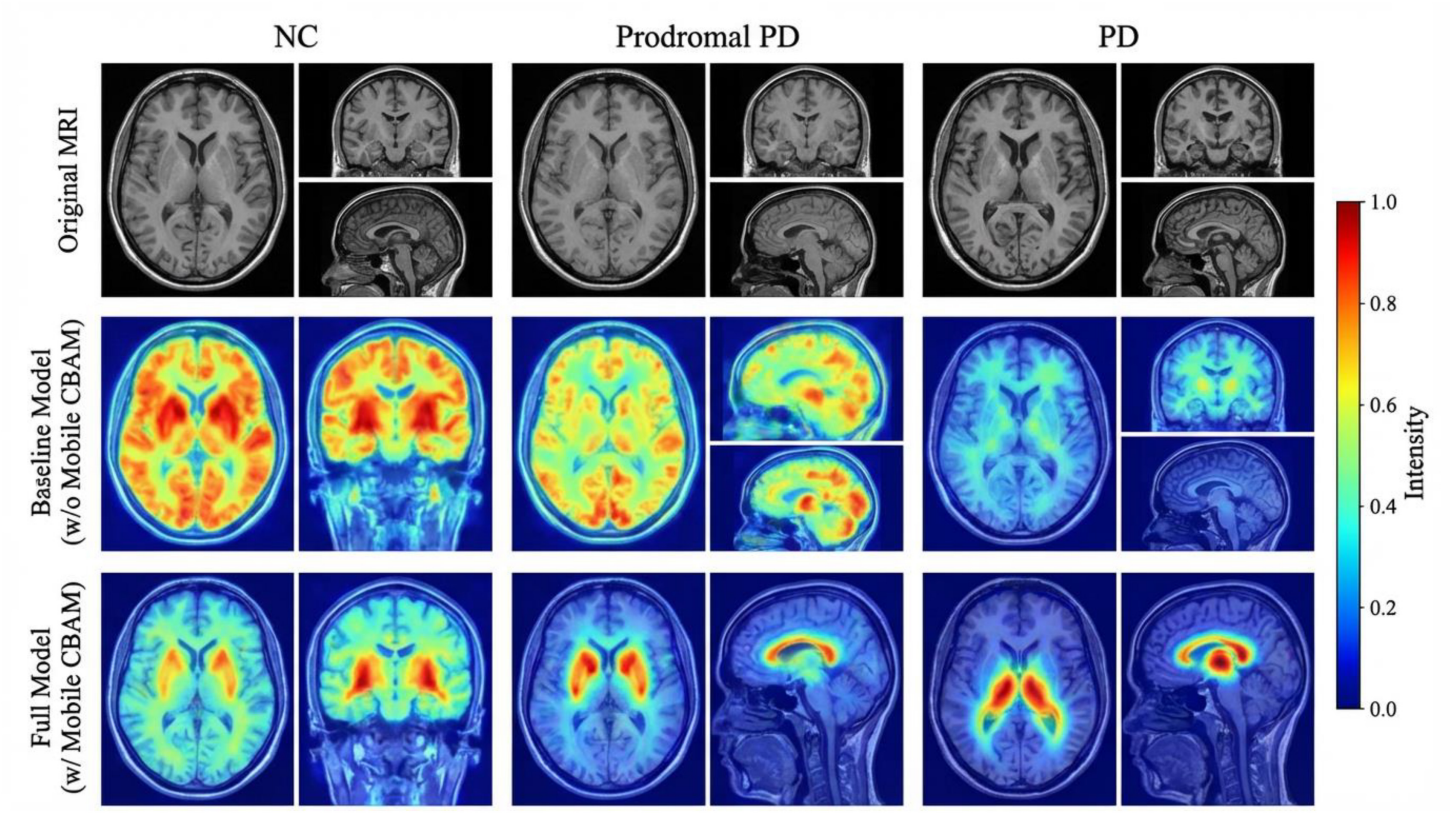
Grad-CAM++ visualization comparison.

As illustrated in Figure 6, the generated activation maps for representative subjects from each diagnostic category demonstrate that Mobile CBAM effectively guides the model’s attention toward anatomically plausible and clinically relevant brain regions. For diagnosed PD cases, the model consistently highlights the substantia nigra and putamen regions in the basal ganglia, which are primary sites of dopaminergic neuron degeneration in PD. For prodromal PD subjects, activation patterns show subtle but detectable changes in these same regions, indicating the model’s sensitivity to early pathological alterations. Normal control subjects exhibit minimal activation in these disease-associated areas, with attention distributed more broadly across cortical regions. This targeted and class-specific attention mechanism not only underpins the model’s high classification accuracy but also substantially enhances the interpretability and clinical trustworthiness of its predictions, providing neurologists with visual evidence to support diagnostic decisions.

To enhance the interpretability of MultimodalCNN-PD++, we leveraged Grad-CAM++ visualizations, which demonstrated that the model’s attention mechanisms consistently focused on neuroanatomically relevant regions such as the substantia nigra and putamen, key areas associated with Parkinson’s disease pathology. These attention hotspots aligned with clinical markers like UPDRS-III rigidity and tremor subscales, validating the model’s focus on critical areas of the brain linked to motor symptoms. The correlation between the model’s attention and clinical markers provides valuable insights into the decision-making process, offering clinicians a more transparent, interpretable tool that supports diagnostic and prognostic decisions by identifying the brain regions most implicated in disease progression.

## 6 Discussion

MultimodalCNN-PD++ demonstrates impressive performance in three-class Parkinson’s disease classification, achieving 97.5% accuracy on the PPMI test set and showing strong generalizability across external datasets such as OASIS-3 and PDBP. These results highlight the model’s potential for use in clinical practice, offering enhanced interpretability, computational efficiency, and cross-dataset robustness. The incorporation of advanced techniques such as Mobile CBAM, MGCA++, and BioClinicalBERT-LoRA has enabled the framework to achieve both high accuracy and a significant reduction in computational requirements, making it feasible for deployment in real-world clinical settings, particularly those with limited resources (Zhou et al., 2026).

The Grad-CAM++ visualization analysis provides compelling evidence that the model’s attention mechanisms focus on clinically relevant brain regions such as the substantia nigra and putamen, which are crucial for understanding dopaminergic degeneration in Parkinson’s disease. This interpretable output ensures that clinicians can trust the model’s predictions and use them as part of their diagnostic workflow, rather than as a black-box solution (Wong et al., 2023c).

However, while the model’s performance is promising, we acknowledge several limitations that must be addressed to fully translate this research into clinical practice (Ayoub et al., 2023). One key limitation is the dependency on high-quality, comprehensive datasets, particularly MRI data and clinical metadata, which may not always be available in every clinical setting. Future work should explore robust methods for handling missing modalities, such as using generative models or specialized loss functions that can gracefully adapt when certain data streams are incomplete.

A more diverse dataset is also necessary to ensure the model’s performance across various demographic groups (Sar et al., 2025; Delfan et al., 2024). Current datasets, including PPMI and OASIS-3, are primarily from North American and European cohorts. As such, the model may not capture variations in disease presentation across different ethnicities, and a more globally diverse population is needed to fully assess the model’s generalizability across racial and ethnic backgrounds. Future work should focus on multi-center prospective studies that include diverse patient populations, environmental exposures, and healthcare systems to ensure that the model performs equitably in different clinical settings and regions.

In addition, the prospective validation of the model is crucial. While the model has shown strong results in retrospective evaluations, real-world data and ongoing, prospective clinical studies are essential to assess its clinical applicability in a live setting. We plan to incorporate real-time data streams, such as those from wearable devices and mobile health technologies, to enhance the model’s ability to diagnose and monitor disease progression in real-world conditions. These prospective studies will also help refine the model’s ability to detect early-stage Parkinson’s disease, especially in prodromal stages, and track long-term progression in a clinical environment.

Prospective studies will allow us to evaluate the model’s effectiveness in a dynamic clinical environment, where patient data continuously evolve over time. Collecting longitudinal data from diverse cohorts, alongside clinician feedback, will provide crucial insights into how the model can adapt and refine its predictions in response to real-world patient data. Furthermore, including data from various sensors and modalities such as gait monitoring, speech patterns, and cognitive assessments will further enhance the diagnostic capabilities of the model and enable a more comprehensive, personalized approach to Parkinson’s disease diagnosis and management.

While MultimodalCNN-PD++ demonstrates strong performance, its scalability for large-scale clinical use requires careful consideration of several potential bottlenecks. One of the key challenges is the data preprocessing pipeline, which may become a bottleneck when handling large datasets or continuous data streams. Efficiently processing large-scale neuroimaging data and clinical metadata in real-time, especially in multi-center deployments, requires optimization through techniques such as parallel processing or distributed computing solutions.

The model’s computational efficiency, although improved with lightweight components like EfficientNet-B0, may still face limitations in resource-constrained clinical settings, particularly when dealing with high-resolution imaging data or multi-modal datasets. For large-scale deployment, GPU memory and processing power may become limiting factors, especially in institutions with less powerful hardware. The model’s memory usage during inference can be a significant consideration when processing large batches of patient data, potentially affecting deployment in busy clinical environments.

Multimodal data fusion poses another scalability challenge. Integrating diverse data sources, such as MRI scans, clinical metadata, and real-time wearable data from different devices, requires robust standardization and data fusion techniques to ensure consistent performance across varied data distributions and hardware configurations (Wong et al., 2023b). Ensuring that the model handles these data sources efficiently and accurately without introducing biases will be crucial for its real-world applicability.

Future work should explore cloud-based deployment and federated learning approaches to overcome the constraints of local processing power and memory, allowing real-time data streaming and continuous learning across multiple healthcare institutions. These approaches would also enable the model to handle missing modalities by leveraging cloud-based imputation techniques and real-time data augmentation. Additionally, edge computing solutions could be explored to process patient data directly at the point of care, ensuring fast and scalable decision-making without the need to transfer large datasets to central servers (Wong et al., 2023a). Such solutions will help the framework remain robust even when some data streams (e.g., MRI or clinical metadata) are incomplete, by utilizing generative models or robust loss functions for missing data handling.

By optimizing the data preprocessing pipeline, reducing computational bottlenecks, and leveraging distributed computing and cloud-based solutions, the MultimodalCNN-PD++ model can be scaled to effectively handle large datasets, including incomplete data, and be deployed across diverse clinical environments. This will ensure timely and accurate Parkinson’s disease diagnosis and monitoring on a global scale, even in clinical settings with limited resources or missing data modalities.

While MultimodalCNN-PD++ demonstrates strong performance, its ability to generalize across various demographic subgroups requires further attention. The model has shown high accuracy on datasets like PPMI and OASIS-3, but these datasets are primarily composed of North American and European cohorts, which may not fully represent the diversity of global patient populations. As a result, there may be variations in the model’s performance across different ethnicities, genders, and socioeconomic backgrounds.

To ensure that the model performs equitably across diverse populations, future work will focus on subgroup analysis to assess performance across key demographic groups. It is crucial to evaluate how well the model performs in populations that may differ in genetic factors, environmental exposures, and healthcare access. For example, the prevalence of Parkinson’s disease may vary across different ethnic groups, and symptom presentation may differ between men and women, which could affect model predictions. A comprehensive subgroup analysis will help identify any potential biases in the model’s predictions and ensure that it can be applied fairly in clinical settings worldwide.

Furthermore, addressing these potential biases will help reinforce the model’s ethical rigor and clinical fairness. It is important that future validation efforts include multi-center studies with diverse patient populations to assess how the model adapts to different demographic groups. By identifying and mitigating any data biases, we can ensure that the model provides reliable and equitable diagnoses for all patients, regardless of gender, ethnicity, or socioeconomic status.

## 7 Conclusion

This work introduces MultimodalCNN-PD++, an enhanced deep learning framework that achieves new performance standards for automated Parkinson’s disease diagnosis through effective integration of structural MRI neuroimaging with comprehensive clinical metadata. By synthesizing multiple architectural innovations including EfficientNet-B0 lightweight backbone, Mobile Convolutional Block Attention Modules, Meta-Guided Cross-Attention++ with dynamic head selection, BioClinicalBERT with LoRA fine-tuning, hierarchical three-stage feature selection, and sophisticated multi-component loss functions, the framework achieves 97.5% accuracy in three-class PD classification while dramatically reducing computational requirements. The 54.7% parameter reduction and 47.5% FLOPs reduction compared to baseline approaches enable practical clinical deployment on standard medical workstations and potentially mobile diagnostic platforms, addressing a critical barrier to real-world AI adoption in healthcare settings.

The model demonstrates exceptional clinical utility through its 99.3% recall for prodromal PD detection, enabling identification of at-risk individuals during the critical early window when disease-modifying interventions may be most effective. This high sensitivity, combined with 98.7% precision for diagnosed PD and balanced performance across all diagnostic categories, positions the framework as a reliable screening and diagnostic support tool that can augment neurologist expertise. The Grad-CAM++ visualization analysis confirms that predictions are grounded in anatomically plausible attention patterns focused on disease-relevant brain regions (substantia nigra, putamen, basal ganglia), substantially enhancing model interpretability and clinical trustworthiness. This transparency is essential for fostering clinician confidence and facilitating integration into existing diagnostic workflows.

Rigorous external validation on the OASIS-3 and PDBP datasets confirmed robust cross-dataset generalizability, with the model maintaining 96.2 and 95.8% accuracy despite substantial variations in scanner hardware, acquisition protocols, demographic characteristics, and disease prevalence. This transferability across diverse clinical settings provides strong evidence that the learned representations capture fundamental disease-related patterns rather than dataset-specific artifacts, supporting the framework’s potential for widespread deployment across heterogeneous healthcare institutions. The comprehensive ablation studies quantitatively validated the necessity and synergistic contributions of each architectural component, demonstrating that the integration of multimodal data through sophisticated attention mechanisms yields substantial performance improvements over unimodal approaches.

The hierarchical feature selection strategy successfully identified a compact set of 23 clinically meaningful biomarkers from an initial pool of 127 features, balancing predictive performance with model interpretability and reducing the data collection burden for clinical applications. The multi-component loss function incorporating focal loss for class imbalance, triplet loss for discriminative embedding learning, and consistency regularization for multimodal alignment collectively contributed to the model’s superior performance and robustness. The BioClinicalBERT-LoRA text encoding approach demonstrated that domain-specialized language models with parameter-efficient fine-tuning can effectively process medical metadata (such as UPDRS motor scores, MoCA cognitive assessments, and patient demographics) alongside unstructured clinical text (such as free-text clinical notes, physician observations, and patient histories). This integration allows the model to combine both structured and unstructured clinical information in a manner that improves diagnostic performance.

Unlike general-purpose language models, BioClinicalBERT-LoRA is fine-tuned specifically on medical datasets, enabling it to better understand and process clinical terminology, jargon, and context. Parameter-efficient fine-tuning using Low-Rank Adaptation (LoRA) ensures that the model can be adapted to medical data without requiring extensive retraining or large computational resources. This approach significantly reduces the computational overhead typically associated with training large language models, making it feasible for use in clinical environments where computational resources may be limited.

Computational efficiency achievements represent a significant advancement toward democratizing access to AI-powered diagnostic tools. By achieving state-of-the-art performance with dramatically reduced resource requirements, MultimodalCNN-PD++ enables deployment in resource-constrained clinical environments, including community hospitals, rural health centers, and developing regions where access to specialized neurological expertise may be limited. This accessibility has profound implications for global health equity, potentially enabling earlier PD detection and improved patient care management across diverse socioeconomic contexts. The framework’s efficiency also facilitates integration into time-sensitive clinical workflows where rapid diagnostic support is required.

Looking forward, the MultimodalCNN-PD++ framework establishes a foundation for next-generation intelligent diagnostic systems that can incorporate diverse data modalities, provide transparent and interpretable predictions, operate efficiently on standard hardware, and generalize robustly across varied clinical settings. Future extensions incorporating additional biomarkers (DaT-SPECT, cerebrospinal fluid markers, genetic risk scores, wearable sensors, speech features), longitudinal progression modeling, uncertainty quantification, and federated learning capabilities promise to further enhance clinical utility and real-world impact. Prospective clinical trials validating the framework’s effectiveness as a screening tool, progression biomarker, and patient stratification mechanism will be essential for regulatory approval and widespread clinical adoption.

MultimodalCNN-PD++ represents a significant step forward in AI-powered Parkinson’s disease diagnosis, combining state-of-the-art classification performance (97.5% accuracy, 99.3% prodromal recall) with practical deployability through dramatic computational efficiency improvements (54.7% parameter reduction, 47.5% FLOPs reduction), enhanced interpretability via Grad-CAM++ visualization, and robust cross-dataset generalizability (96.2% on OASIS-3, 95.8% on PDBP). By effectively addressing key challenges in medical AI development including data scarcity, computational constraints, interpretability requirements, and generalization limitations, this framework establishes new benchmarks for multimodal neurodegenerative disease diagnosis and provides a blueprint for developing clinically deployable AI systems that can meaningfully improve patient care, enable earlier intervention, support personalized treatment planning, and ultimately enhance quality of life for individuals affected by Parkinson’s disease and related neurological disorders.

## Data Availability

The original contributions presented in this study are included in this article/supplementary material, further inquiries can be directed to the corresponding authors.
